# Guest edited collection serological study of SARS-CoV-2 antibodies in japanese cats using protein-A/G-based ELISA

**DOI:** 10.1186/s12917-022-03527-7

**Published:** 2022-12-21

**Authors:** Ichiro Imanishi, Ryota Asahina, Shunji Hayashi, Jumpei Uchiyama, Masaharu Hisasue, Masahiro Yamasaki, Yoshiteru Murata, Shigeru Morikawa, Tetsuya Mizutani, Masahiro Sakaguchi

**Affiliations:** 1 Department of Microbiology, Kitasato University School of Medicine, 1-15-1 Kitasato, Minami-ku Sagamihara-shi, Kanagawa, Kanagawa Japan; 2 Faculty of Medicine, Department of Dermatology, Kyoto University, Kyoto, Japan; 3 Department of Bacteriology, Graduate School of Medicine Dentistry and Pharmaceutical Sciences, Okayama University, Okayama, Japan; 4 Center for Human and Animal Symbiosis Science, Azabu University, Kanagawa, Japan; 5 Department of Veterinary Internal Medicine, Iwate University, Iwate, Japan; 6 Research and Education Center for Prevention of Global Infectious Diseases of Animals, Tokyo University of Agriculture and Technology, Tokyo, Japan; 7 Murata Animal Hospital, Chiba, Japan; 8 Faculty of Veterinary Medicine, Department of Microbiology, Okayama University of Science, Ehime, Japan; 9 Institute of Tokyo Environmental Allergy, Tokyo, Japan

**Keywords:** SARS-CoV-2, Seroepidemiological studies, Cats, Health risk behaviours

## Abstract

**Background:**

Little is known about the epidemic status of severe acute respiratory syndrome coronavirus 2 (SARS-CoV-2) in cats in Japan due to insufficiently reliable seroepidemiological analysis methods that are easy to use in cats.

**Results:**

We developed a protein-A/G-based enzyme-linked immunosorbent assay (ELISA) to detect antibodies against SARS-CoV-2 in cats. The assay was standardized using positive rabbit antibodies against SARS-CoV-2. The ELISA results were consistent with those of a conventional anti-feline-immunoglobulin-G (IgG)-based ELISA. To test the protein-A/G-based ELISA, we collected blood samples from 1,969 cats that had been taken to veterinary clinics in Japan from June to July 2020 and determined the presence of anti-SARS-CoV-2 antibodies. Nine cats were found to have SARS-CoV-2 S1-specific IgG, of which 4 had recombinant receptor-binding domain-specific IgG. Of those 9 samples, one showed neutralizing activity. Based on these findings, we estimated that the prevalence of SARS-CoV-2 neutralizing antibodies in cats in Japan was 0.05% (1/1,969 samples). This prevalence was consistent with the prevalence of neutralizing antibodies against SARS-CoV-2 in humans in Japan according to research conducted at that time.

**Conclusions:**

Protein-A/G-based ELISA has the potential to be a standardized method for measuring anti-SARS-CoV-2 antibodies in cats. The infection status of SARS-CoV-2 in cats in Japan might be linked to that in humans.

**Supplementary Information:**

The online version contains supplementary material available at 10.1186/s12917-022-03527-7.

## Background

Coronavirus disease 2019 (COVID-19) caused by severe acute respiratory syndrome coronavirus 2 (SARS-CoV-2) has spread worldwide and remains a pandemic [1]. SARS-CoV-2 infections have become widespread [2], even in vaccinated individuals [3, 4]. Minimizing exposure to SARS-CoV-2 is crucial to curtail the COVID-19 pandemic. The main route of transmission of SARS-CoV-2 is thought to be the adhesion of droplets from infected people to the respiratory mucosa of others [5]. Previous reports revealed that mink with SARS-CoV-2 infections can release the virus into the air [6], and that deer are exposed to multiple SARS-CoV-2 variants from humans and are capable of sustaining transmission in nature [7]. Although animals may become a reservoir for SARS-CoV-2 infection from which recombinant viruses could possibly emerge, transmission through animals has not been emphasized to date.

Several cases of confirmed SARS-CoV-2 infection in cats and dogs have been reported [8]. As of 10 November 2021, all of these infections have been presumed to have been transmitted from humans, and there have been no reported transmission cases from cats or dogs to humans [9]. However, cats may be more susceptible to SARS-CoV-2 than other animal species [10], and laboratory transmission experiments have shown that cats can transmit droplet or airborne infections to other cats [11]. Additionally, cats have been shown to induce the emergence of SARS-CoV-2 variants [12] that might induce breakthrough infections. Because cats are one of the most common companion pets worldwide, studies on the prevalence of SARS-CoV-2 infection in cats are critical.

Until vaccination began, the prevalence of SARS-CoV-2 neutralizing antibodies was used to estimate the cumulative number of COVID-19 cases, because SARS-CoV-2 neutralizing antibodies are reported to persist for at least 3 months once someone is infected [13]. A vaccine against SARS-CoV-2 for cats has been developed [14, 15], but as of July 2020, there were no vaccinated cats to interfere with seroepidemiological studies, and the prevalence of SARS-CoV-2 neutralizing antibodies could be used at that time to estimate the cumulative number of SARS-CoV-2 infections in cats. However, the seroprevalence of SARS-CoV-2 antibodies in cats in various countries is limited, and to our knowledge, no information for Japan has been reported to date.

Therefore, in the present study, we sought to estimate the cumulative number of SARS-CoV-2 infections in cats in Japan as of July 2020. We developed an enzyme-linked immunosorbent assay (ELISA) that can measure anti-SARS-CoV-2 antibodies and conducted ELISAs using serum or plasma from cats that had been taken to 101 veterinary clinics in Japan from 1 June to 31 July 2020. We determined the seroprevalence of SARS-CoV-2 antibodies by performing neutralizing antibody tests on ELISA-positive samples. We also surveyed the owners of cats in the sample to determine the housing and husbandry of the cats with SARS-CoV-2 neutralizing antibodies.

## Results

### Samples collected from 1 to 2020 to 31 July 2020, in Japan

Before we collected the samples, the necessary sample size was determined. At the time, only one report, from Wuhan, China, was available for the seroprevalence of SARS-CoV-2 immunoglobulin G (IgG) in cats [16]. According to this Chinese report, the seroprevalence of SARS-CoV-2 IgG in cats in veterinary clinics was 8.70% [4/52 samples, January–March 2020, 95% confidence interval (CI): 2.90–20.86%] [16]. Referring to the cumulative COVID-19 cases per 1,000 people in Japan and Wuhan, China, at that time from the World Health Organization database (https://covid19.who.int/accessed: 25th May 2020), the prevalence of SARS-CoV-2 infection in cats was assumed to be similar in these countries. Thus, the assumed seroprevalence in Japan was tentatively set to 3.00% for the sample size calculation. Based on this seroprevalence assumption, the sample size was calculated to be 1,562 cases (α error = 0.05, β error = 0.80) using goodness-of-fit tests. Considering the loss of samples because of a lack of information (i.e., clinical history and cat husbandry), in the present study we included the available 1,969 cat blood samples collected from 1 to 2020 to 31 July 31, 2020 (Table 1).

With the consent of the cat owners, samples were collected by veterinarians at 101 veterinary clinics in Japan from June 1, 2020, to July 31, 2020. The sampling period was set at 2 months after the virus outbreak, when the second wave of infections occurred in Japan (Supplementary Fig. 1).

**Table 1:** Cat blood samples were collected in Japan from 1 June to 31 July, 2020

	Prefecture								Total
	**Tokyo**	**Chiba**	**Kanagawa**	**Osaka**	**Kyoto**	**Aichi**	**Shizuoka**	**Iwate**	
**No. veterinary clinics**	20	30	19	5	13	7	6	1	101
**No. samples**	420	479	317	42	235	253	208	15	1,969
Serum	277	369	275	35	213	173	151	15	1,508
Plasma	143	110	42	7	22	80	57	0	461
**Age** **(mean ± SD)**	8.06 ± 4.69	7.47 ± 5.30	8.61 ± 5.06	7.03 ± 4.55	6.75 ± 4.48	7.70 ± 4.50	7.94 ± 4.67	10.09 ± 5.95	7.70 ± 4.93
**Sex**									
Male	33	46	17	8	19	37	18	3	181
Castrated male	182	191	148	18	94	71	93	6	803
Female	33	46	19	3	32	54	18	1	206
Spayed female	172	196	133	13	90	91	79	5	779
**Animal holding size**									
Single	277	278	186	24	126	148	113	9	1,161
Multiple	134	182	131	18	99	102	88	6	760
Unknown	9	19	0	0	10	3	7	0	48
**Housing conditions**									
Indoors only	396	409	291	40	208	190	167	13	1,714
Indoors–outdoors	19	38	22	2	13	19	26	2	141
Outdoors only	5	32	4	0	14	44	15	0	114
**Clinical sign**									
High fever (≥ 39.0 °C)	103	23	17	1	5	11	4	2	166
Respiratory clinical signs	29	25	21	0	9	13	7	5	109
GI clinical signs	50	63	43	4	25	35	18	6	244

*GI Gastrointestinal

The gastrointestinal symptoms included abdominal pain, belching, stomachache, bloating, loss of appetite, vomiting, abdominal pain, constipation, diarrhoea, and/or melena.

### Cat housing and husbandry

Using a questionnaire, we asked the owners about the housing and husbandry of their cats, including housing indoors only, free access outside, and living outdoors only, neutering, and multiple animals owned (Table 1). The primary housing environment for the cats was indoors only (approximately 88%). The prevalence of neutering was approximately 80%. The prevalence of multiple cats owned was approximately 40%. In the present study, 114 cats living outdoors were sampled in trap-neuter-return programs. No cats were living in a shelter or cattery.

### Development of an ELISA using protein-A/G conjugated with horseradish peroxidase (protein-A/G-based ELISA)

**Fig. 1:**
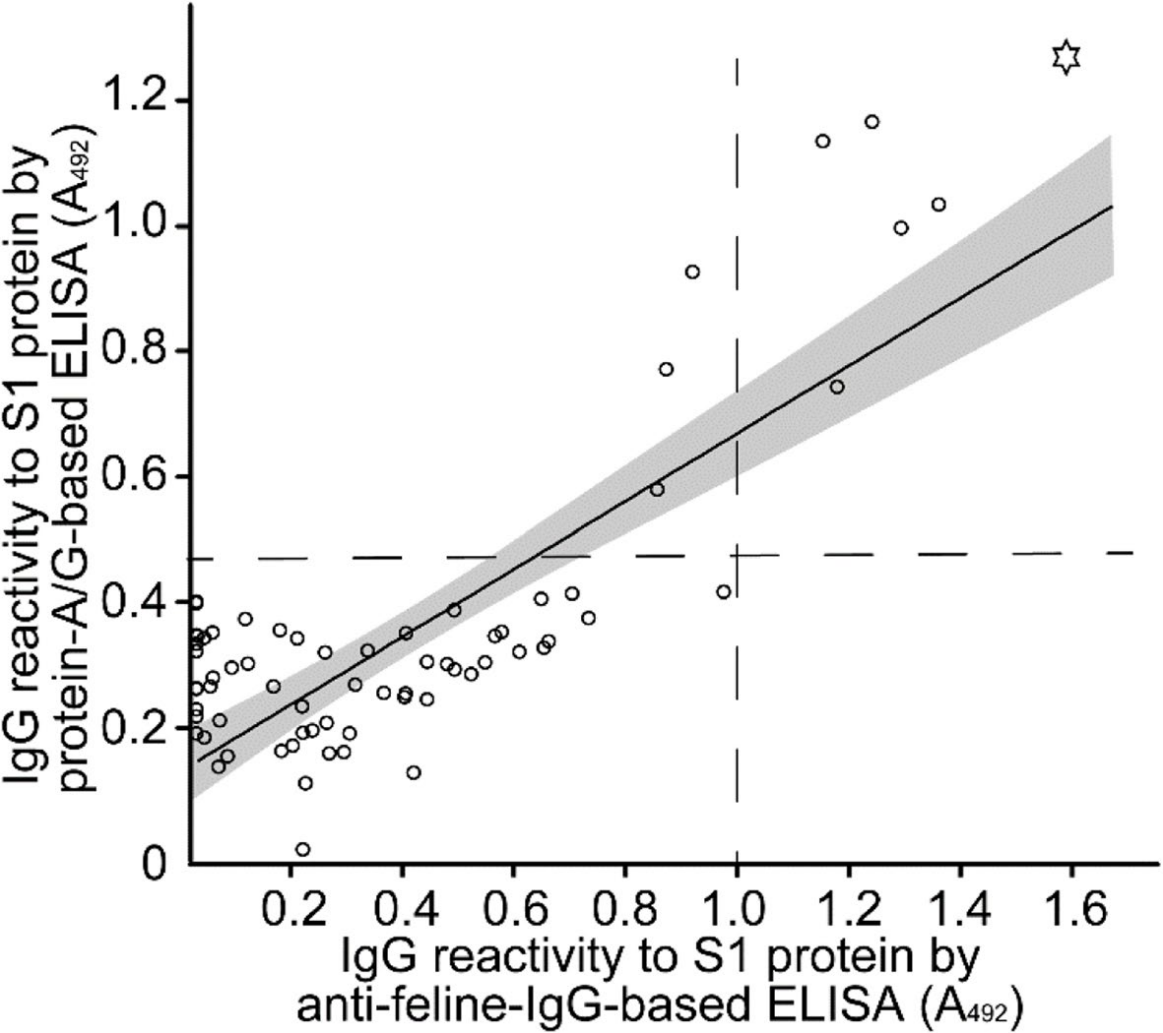
Reliability of an ELISA using protein-A/G conjugated with horseradish peroxidase (protein-A/G-based ELISA). The correlation of antibody reactivity to the S1 protein, as assessed by protein-A/G-based ELISA, with that assessed by anti-feline-IgG-based ELISA is shown. The absorbance at 492 mm (A_492_) was measured in both ELISAs. A_492_ of protein-A/G-based ELISA was normalized to positive control rabbit sera. The cut-off was set as the mean value + 3 standard deviations (SDs) of negative control samples, which is shown as a dashed line. Samples with a higher value than the cut-off value were considered positive. The regression line and 95% confidence intervals are indicated by the black line and grey area, respectively. The circles indicate values for individual tested samples. The star indicates a sample with SARS-CoV-2 neutralizing activity

We confirmed that a gene fusion of the Fc-binding domain of protein A and protein G (protein-A/G) conjugated with horseradish peroxidase reacts strongly to cat IgG and rabbit IgG, but weakly to cat IgM and not to bovine serum albumin (Supplementary Fig. 2). The accuracy of the protein-A/G-based ELISA was validated by confirming that the correlation of IgG reactivity to the S1 protein was similar to that of conventional ELISA using anti-feline IgG conjugated with horseradish peroxidase as the detecting antibody. We examined 34 samples, namely, 9 positive and 25 negative samples, and 162 negative control samples via protein-A/G-based ELISA, which detected the anti-S1-protein antibody (Fig. 1). A comparison of the antibody reactivity measured by protein-A/G-based ELISA with that measured by anti-feline-IgG-based ELISA revealed a strong correlation (*r*^2^ = 0.83 and *p* < 0.0001).

### Seroprevalence of anti-SARS-CoV-2 antibodies in cats in Japan

**Fig. 2:**
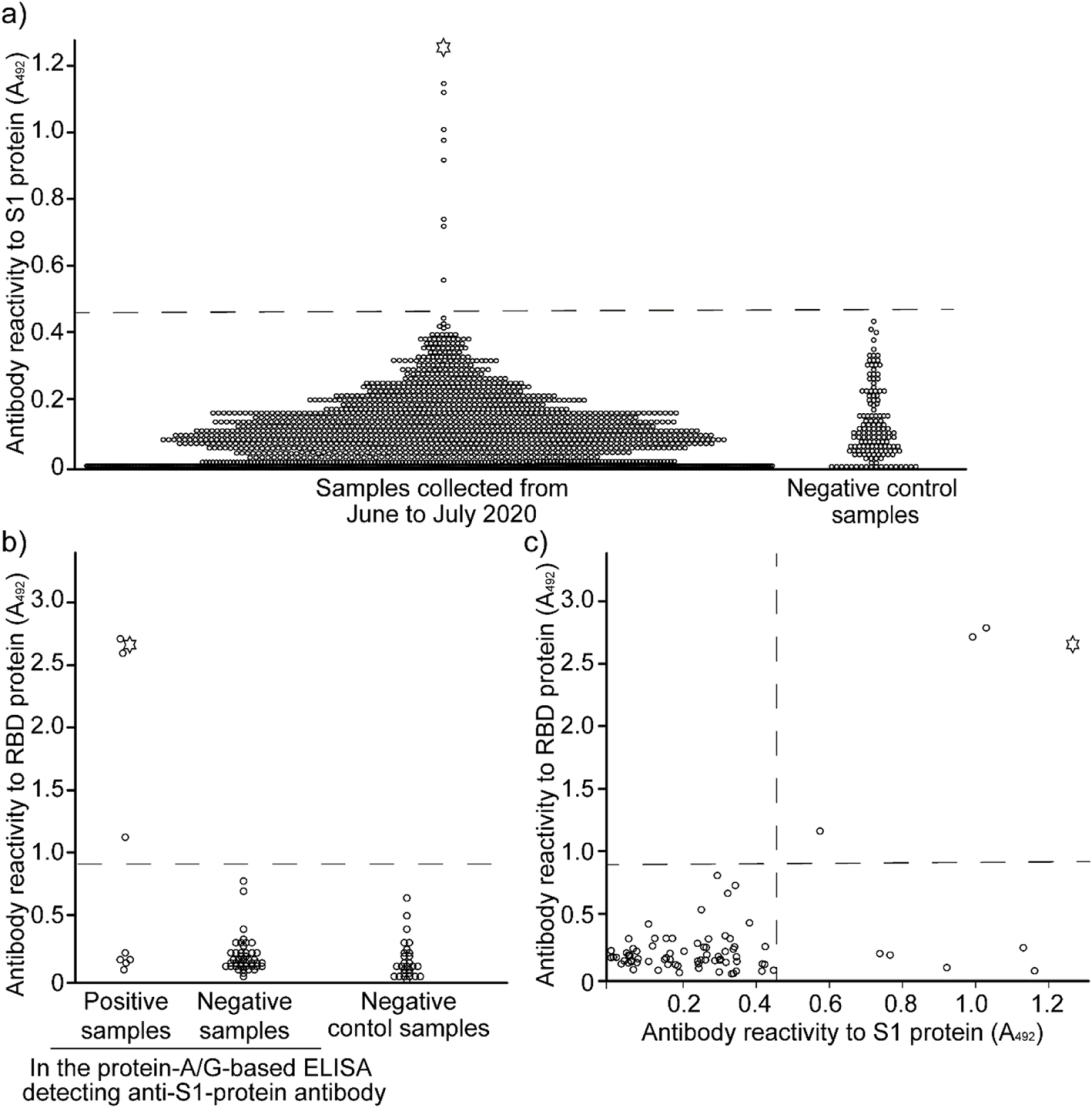
Antibody reactivity to SARS-CoV-2 proteins in cats in Japan. Antibody reactivity was measured by an ELISA using protein-A/G conjugated with horseradish peroxidase (protein-A/G-based ELISA). The absorbance at 492 mm (A_492_) was normalized to that for positive control rabbit sera. Samples with a value higher than the cut-off were considered positive. The circles indicate individual tested samples. The star indicates a sample showing SARS-CoV-2 neutralizing activity in a neutralizing antibody assay. **a** Antibody reactivity to the S1 protein among 1,969 samples. The mean A_492_ ± SD of 145 negative control samples was 0.133 ± 0.111 A_492_, and the cut-off value (mean + 3 SDs) was 0.469 A_492_, shown as a dotted line. **b** Antibody reactivity to the RBD protein in 9 samples that were positive for anti-S1-protein antibody. The mean A_492_ ± SD of 28 negative control samples without specific selection was 0.231 ± 0.267 A_492_, and the cut-off (mean + 3 SDs) was 1.033 A_492_, shown as the dotted line. We screened 75 samples, namely, 9 positive samples and 66 negative samples in the protein-A/G-based ELISA, detecting anti-S1-protein antibody. **c** Comparison of antibody reactivities with those to the S1 protein, as measured by protein-A/G-based ELISA. Among the 9 samples positive in the protein-A/G-based ELISA detecting anti-S1-protein antibodies, 4 cats showed antibody reactivity to the RBD protein

The screening of the 1,969 samples by ELISA for the S1 protein identified 9 samples with antibody reactivity (seroprevalence: 0.46%) (Fig. 2a). In addition, ELISA of these 9 positive samples to detect the anti-receptor-binding domain (RBD) protein antibody revealed 4 samples with antibody reactivity (seroprevalence: 0.20%) (Fig. 2b). Comparing antibody reactivity to the S1 protein with that to the RBD protein showed that these 4 samples had antibodies with high reactivity to both proteins (Fig. 2c).

Because an ELISA can produce false-positive results owing to antibody cross-reactivity among coronaviruses [17], the SARS-CoV-2 neutralizing activity of these 9 positive samples was determined in vitro. One sample neutralized SARS-CoV-2 when diluted up to 1:80, whereas the remaining 8 samples (diluted up to 1:20) did not (Table 2). We concluded that those 8 ELISA positive samples were false-positive results because the positive and negative predictive value of the virus neutralization test was 1.00 in 1 rabbit positive sample (No. 40,592-R001, Sino Biological, Beijing, China) and 14 negative samples (Supplementary Table 1). The sample that neutralized SARS-CoV-2 was from a cat with no respiratory or gastrointestinal clinical signs or high fever in the previous 3 months. It was kept indoors only and had no history of escape from the house (Table 2). Accordingly, the seroprevalence of SARS-CoV-2 antibodies in cats in Japan was assumed to be 0.05% (1/1,969 samples). We found no significant difference in the percentage of SARS-CoV-2 neutralizing antibodies in humans [0.10%, 8/7,980 samples, 1–7 June 2020, Japan] [18] or in cats in this study (Fisher’s exact test, *p* = 0.44).

**Table 2:** Information on the nine cats with positive results in the protein-A/G-based ELISA, detecting anti-S1-protein IgG.

Sample No.^a^	Sampling date (2020)	Sample type	Age	Breed	Sex^b^	Living environ -ment	Multiple cats owned	Clinical sign			Protein-A/G-based ELISA targeted:		Neutralizing Ab titre
								**High fever** ^c^	**Respir** ^d^	**GI** ^e^	**S1 (A** ^492^ **)**	**RBD (A** ^492^ **)**	
○1	11th July	Serum	9y11mo	Mixed	M	Indoor-only	-	-	-	-	1.25	2.65	1:80
2	21st June	Serum	2y5mo	Somali	MC	Indoor-only	-	-	-	-	1.15	0.06	< 1:20
3	19th June	Serum	8y9mo	Mixed	MC	Indoor-only	-	-	-	-	1.12	0.25	< 1:20
4	29th July	Serum	10y3mo	Mixed	FS	Indoor-only	-	-	-	-	1.02	2.78	< 1:20
5	1st July	Serum	9mo	Bengal	F	Indoor-only	-	-	-	-	0.98	2.70	< 1:20
6	19th July	Plasma	7mo	Mixed	FS	Indoor-only	-	+	-	-	0.91	0.09	< 1:20
7	18th July	Serum	1y11mo	Mixed	F	Indoor-only	-	-	-	-	0.76	0.19	< 1:20
8	17th July	Plasma	10y4mo	Mixed	F	Free-access-outside	-	-	+	-	0.73	0.20	< 1:20
9	4th July	Serum	8y5mo	Munch-kin	MC	Indoor-only	-	-	-	-	0.57	1.17	< 1:20

^a^Sample No. 1 contained neutralizing antibody to SARS-CoV-2, which is shown as a circle. Sample Nos. 1–4 showed positive results in the ELISA detecting anti-RBD-protein antibody, which are underlined.

^b^*MC* Male castrated, *FS* Female spayed.

^c^High fever was defined as a body temperature above 39 °C.

^d^Respiratory symptoms included cough, sneezing, conjunctivitis, and nasal and/or ocular discharge.

^e^GI, gastrointestinal. Gastrointestinal symptoms included abdominal pain, belching, stomachache, bloating, loss of appetite, vomiting, abdominal pain, constipation, diarrhoea, and/or melena.

## Discussion

To determine the situation of SARS-CoV-2 infection in cats, we collected blood samples from 1,969 cats that had been taken to veterinary clinics in Japan in June and July 2020 to determine the presence of SARS-CoV-2-specific antibodies. Of these samples, one showed neutralizing activity (seroprevalence: 0.05%, 1/1,969 samples), which was not significantly different from the concurrent human seropositivity prevalence [18]. This suggests that the prevalence of SARS-CoV-2 infection in cats may be associated with the COVID-19 epidemic in humans.

We performed an ELISA for S1 and RBD of SARS-CoV-2 followed by a neutralization test. Although seroepidemiological studies targeting the nucleocapsid of SARS-CoV-2 have been conducted [14, 17, 19, 20], the anti-SARS-CoV-2 nucleocapsid-protein specific IgG cross-reacted with those of feline coronavirus (FCoV) [20] and other coronaviruses [21]. In contrast, anti-SARS-CoV-2 RBD IgG rarely cross-reacts with the RBD of feline coronaviruses [16, 22], although it has been suggested that an ELISA targeting SARS-CoV-2 RBD risks failing to detect samples with neutralizing activity [14]. S1 is an RBD-containing spike protein that reacts strongly to antibodies against SARS-CoV-2, and has a unique structure in each coronavirus [23]. To date, no cases showing cross-reactivity of cat S1-specific antibodies derived from SARS-CoV-2 with other coronaviruses, such as FCoV have been reported [17, 20, 24]. An analysis of antibody titres in the blood of human COVID-19 patients revealed that measurement of IgG against spike proteins, especially S1 and RBD, correlates with the presence of neutralizing antibodies and is an excellent indicator of past infection [25]. Because identification of the presence of a neutralizing antibody requires special laboratories equipped with biosafety level 3 facilities and involves the risk of analysing multiple samples [26], screening for the presence of S1- and RBD-specific antibodies, and then confirming the presence of neutralizing antibodies in SARS-CoV-2-specific antibody-bearing samples is currently considered an appropriate identification strategy.

In previous reports on ELISA for determining the presence of feline anti-SARS-CoV-2 IgG, an anti-feline IgG antibody was used as the detecting antibody [16, 22, 27–29]. In SARS-CoV-2 specific antibody-bearing samples are currently considered an appropriate identification standard. As of 30 September 2021, no feline anti-SARS-CoV-2 IgG antibodies, which can be used as a positive control, were commercially available. Protein-A/G-based ELISAs have been used to investigate infectious diseases across animal species [30, 31]. The detection system developed in the present study can be applied in many laboratories because rabbit anti-SARS-CoV-2 antibodies are commercially available. We cannot ignore that protein-A/G also detects a small amount of feline IgM. Unlike IgG, the blood levels of IgM decrease within 2 weeks after infection [32], and IgM has been reported to be less elevated than IgG in cats infected with SARS-CoV-2 [33]. Furthermore, we showed that the protein-A/G-based ELISA results were consistent with the anti-feline-IgG-based ELISA results, as shown in Fig. 1 and Supplementary Fig. 3. Therefore, although we cannot deny the possibility of detecting IgM in cats with protein-A/G-based ELISA, the assay is more likely to detect IgG in cats.

Here, we used a virus neutralization test that has been supported by many studies [16, 22, 26]. The sensitivity and specificity of the virus neutralization test in cats remains unknown. The surrogate virus neutralization test for SARS-CoV-2 in humans overestimates the concentration of low neutralizing antibodies in plasma [34]. This high positivity rate could be attributed to substances such as heparin in the plasma inhibiting the virus [35]. Therefore, we used the plaque reduction assay. The inactivated viral samples used in the present study were serum samples, and none of the samples used as negative controls were detected as false positives. Therefore, the percentage of cats with neutralizing antibodies against SARS-CoV-2 that we obtained may reflect the rate of cumulative SAR-CoV-2 infections in cats.

Similarities in seroprevalence between humans and cats have been observed in Japan. This suggests that the opportunity for SARS-CoV-2 infection in cats is influenced by the extent of COVID-19 prevalence in humans. Cats kept by COVID-19 patients or people with a history of COVID-19 have a high probability of being infected with SARS-CoV-2 [29]. The prevalence obtained in the present study is supported by these reports.

Experimental data from cat infections showed that neutralizing antibody levels increase within 7 days of infection and are retained for at least 42 days [33]. SARS-CoV-2 can be inactivated by ultraviolet light, so it has been shown to spread more in winter than in summer months, when there is more sunlight [36]. Therefore, if the survey is conducted again in winter, more cats are expected to have neutralizing antibodies. Variants such as the omicron SARS-CoV-2 variant prevalent in January 2022 may be more infectious than the SARS-CoV-2 variant prevalent in cats in 2020 [37], and the transmission situation may have changed. The prevalence of infection in cats in 2022 may be more serious, and follow-up research would be interesting.

A survey regarding the prevalence of neutralizing antibodies in cats admitted to veterinary clinics in 2020 has been conducted in various countries, with positive rates varying from 0 to 7.14% (mean ± SD: 3.42% ± 2.78%) [14, 16, 22, 26–29, 38, 39]. Because most of these epidemiological surveys do not mention cat housing or husbandry, we were unable to examine the factors associated with the spread of SARS-CoV-2 infection by comparing other reports with the present study. Because of the variability of the prevalence in cats, even when human infection status was considered, we believe that there are factors in cat housing and husbandry that contribute to the spread of SARS-CoV-2 infection. Future epidemiological surveys should include detailed descriptions of cat housing and husbandry. This will facilitate searching for factors involved in the spread of SARS-CoV-2 infection. There are several limitations to the present study. We identified one cat with neutralizing antibodies. Due to ethical constraints, we were unable to interview the cat’s owners about their COVID-19 history. As a result, it is challenging to ascertain how the cat became infected and if it infected the human.

As of July 2020, 0.05% of cats in Japan had a history of infection with SARS-CoV-2, which was similar to the prevalence in humans. Because cats are companion animals, the infection status of SARS-CoV-2 is considered to be linked to that in humans. We have developed an ELISA for SARS-CoV-2 antibody screening in cats that can be reproduced virtually anywhere in the world.

## Methods

### Collection of samples with clinical history and data pertaining to cat housing and husbandry

According to a literature review on 25 May 2020, the seroprevalence in cats was assumed to be the lower limit of a 95% CI of the Wuhan report [7.69%, 4/52 samples, January–March 2020] [16]. The sample size was calculated based on the seroprevalence using G*Power 3.1.9.6 (downloaded from https://www.psychologie.hhu.de/arbeitsgruppen/allgemeine-psychologie-und-arbeitspsychologie/gpower on 25 April 2020).

With the consent of the cat owners, veterinarians collected blood samples from cats admitted to 101 veterinary clinics in Japan from June 1 to July 31, 2020. The sampling period was set at 2 months after the virus outbreak, when the second wave of infections occurred in Japan (Supplementary Fig. 1). Blood samples were collected in 4 mL vacutainer blood collection tubes containing coagulation accelerator and serum-separating agent, or heparin/ethylenediaminetetraacetic acid disodium salt dihydrate (Vebnoject II, Terumo, Japan). Serum or plasma was separated using a centrifuge at each veterinary clinic, transferred into 1.5 mL tubes, and transported to Kitasato University at − 20 °C. The transported samples were stored at − 80 °C until use.

Veterinarians completed a questionnaire on the clinical history of the animals within 3 months of blood collection. Cat owners visiting veterinary clinics were surveyed about cat housing and husbandry aspects, such as living environment, neutering, and multiple animals owned. Housing was categorized into 3 types: indoors only, free access outside, and living outdoors (i.e., stray cats and feral cats). As negative controls, we also used cat blood samples collected from 1 to 2015 to 31 March 2015 for the Azabu University bioresource banking project, which were stored at -80 °C until use. This study was carried out in compliance with the ARRIVE 2.0 guidelines and was approved by the Animal Ethics Committee of Azabu University (No. 210,407–7).

### Detection methods for anti-SARS-CoV-2 antibodies in feline serum/plasma samples

An ELISA using protein-A/G conjugated with horseradish peroxidase was used to detect the anti-S1-protein antibody or anti-RBD-protein antibody [30]. Based on previous reports [16, 22, 40], 100 ng per well of recombinant S1 protein (No. S1N-C52H3, Acro Biosystems, Newark, DE, USA) and 50 ng per well of RBD protein (No. 230–30,162–100; Ray Biotech, Peachtree Corners, GA, USA), which were derived from the 2019-nCoV strain, were diluted in 50 mM carbonate buffer with 100 µL solution to coat half-well plates (Costar, Washington, DC, USA). All plates were incubated overnight at 4 °C and washed once, and nonspecific binding sites were blocked with 0.5% bovine serum albumin (Merck, Darmstadt, Germany) in phosphate-buffered saline (PBS) containing 0.05% Tween-20 (PBS-T) at 25 °C for 1 h. After washing 3 times, 100 µl of diluted sample (1:100) was added to the appropriate well and incubated at 25 °C for 1 h. To reduce the nonspecific reaction of blood, samples were diluted in PBS-T containing 5% skim milk (Fujifilm Wako Pure Chemical Corporation, Osaka, Japan) and 10% foetal bovine serum (FBS) (Bio West, Riverside, MO, USA) and prereacted at 37 °C for 1 h. The plates were washed 3 times and incubated with protein-A/G conjugated with horseradish peroxidase, which was diluted in PBS-T (1:25,000; Thermo Fisher Scientific, Waltham, MI, USA) at 25 °C for 1 h. After washing 3 times, the plates were incubated with *o*-phenylenediamine dihydrochloride (Sigma Aldrich) at 25 °C for 30 min. The reaction was terminated by adding 4 N H_2_SO_4_. The absorbance at 492 mm (A_492_) was measured using a spectrophotometer (Multiskan JX; Thermo Fisher Scientific).

An anti-S1 rabbit polyclonal antibody (1:100; No. GTX135356, Genetex, Irvine, CA, USA) and an anti-RBD rabbit monoclonal antibody (1:80,000; No. 40,592-R001, Sino Biological) were used as positive controls. IgG reactivity in the positive controls was adjusted to 1.0 A_492_. The samples of the positive control serially diluted to 1:2, 1:4, and 1:8 from the dilution condition of 1.0 A_492_ were adjusted to 5, 2.5, and 1.25 A_492_, respectively. Rabbit plasma (Sigma Aldrich) diluted 1:100 was used as a negative control.

All samples were assayed in parallel on the same plate. For serum samples showing values higher than the range of detection, an additional 3 serial dilutions were assayed. All experiments were performed in triplicate. PBS-T was used in all washes. The cut-off was set as the negative control sample mean ± 3 SD. Samples with a value higher than the cut-off were considered positive.

Conventional ELISAs using anti-feline IgG as the detecting antibody were performed as described by Zhao et al. [20]. The antigens were 100 ng per well of recombinant S1 protein (No. S1N-C52H3, Acro Biosystems) and 50 ng per well of RBD protein (No. 230–30,162–100; Ray Biotech). As a secondary antibody, we used horseradish peroxidase-conjugated goat anti-feline IgG (H + L) (1/1,000). We examined 34 samples collected from June to July 2020, namely, 9 positive and 25 negative samples in the protein-A/G-based ELISA, detecting an anti-S1-protein antibody, and 162 negative control samples (Fig. 1). The dilution reagent and reaction times were the same as those for protein-A/G-based ELISA.

The neutralization activity of diluted samples to SARS-CoV-2 virus was examined by a test described elsewhere [41]. Briefly, samples were heat-inactivated through incubation at 56 °C for 30 min. Each sample was serially diluted twofold with Dulbecco’s modified Eagle’s medium (DMEM), according to the absorbance value. The diluted sample was mixed with an equal volume of diluted virus containing 100 plaque forming units (SARS-CoV-2 strain 2019-nCoVJPN/TY/WK-521/2020) and incubated at 37 °C for 1 h. VeroE6/TMPRSS cells in 24-well plates were inoculated with the sera-virus mixture at 37 °C for 1 h. Plaque forming units were calculated by the method described elsewhere [42]. Subsequently, the mixture was replaced with DMEM containing 2.5% FBS and 0.8% carboxymethylcellulose. After 3 days of culture, the cells were fixed to the plates with 8% paraformaldehyde and stained with 0.5% crystal violet. All the samples were tested in triplicate, and a sample dilution titre that resulted in a plaque reduction by at least 50% was defined as the neutralization titre. Each sample was diluted up to 1:20. To verify the reliability of the virus neutralization test, we used an anti-RBD rabbit monoclonal antibody (No. 40,592-R001, Sino Biological) as a positive control, and 14 samples were used as negative controls (Supplementary Table 1).

### Statistical analysis

The correlation of antibody reactivity to the S1 protein, as assessed by protein-A/G-based ELISA, with that assessed via conventional ELISA using anti-feline IgG conjugated with horseradish peroxidase (anti-feline-IgG-based ELISA) was analysed using a Pearson correlation coefficient test.

The prevalence of the anti-SARS-CoV-2 antibody in cats and humans was compared using Fisher’s exact test. Significance was set at *p* < 0.05. All statistical analyses were performed using GraphPad Prism 9.0 (GraphPad Software Inc., San Diego, CA, USA).

## Supplementary Information

**Additional file 1**: **Table 1**. Information on the 14 cats as negative controls in the neutralization test. 

**Additional file 2**: **Figure 1**.Transition of COVID-19 cases from 16 January to 31 August, 2020 reported in the national surveillance in Japan. 

**Additional file 3**: **Figure 2**. Binding ability of protein-A/G conjugated with horse radish peroxidase to feline IgG and IgM, and rabbit IgG.

**Additional file 4**: **Figure 3**. Correlation of IgG reactivities obtained by protein-A/G-based ELISA with those obtained by anti-feline-IgG-based ELISA.

## Data Availability

All data generated or analysed during this study are included in this published article and its supplementary information files.

## Abbreviations

COVID-19: Coronavirus disease 2019
CI: Confidence interval
DMEM: Dulbecco’s modified Eagle’s medium
ELISA: Enzyme-linked immunosorbent assay
FBS: Foetal bovine serum
Ig: Immunoglobulin
PBS: Phosphate-buffered saline
PBS-T: PBS containing 0.05% Tween-20
Protein-A/G: Gene fusion of the Fc-binding domain of protein A and protein G
RBD: Recombinant receptor-binding domain
SARS-CoV-2: Severe acute respiratory syndrome coronavirus 2
SD: Standard deviation
