# Supplementary material for: Guest edited collection serological study of SARS-CoV-2 antibodies in japanese cats using protein-A/G-based ELISA
Source: BMC Vet Res. 2022 Dec 21;18:443. doi: 10.1186/s12917-022-03527-7 (PMC9767852; doi:10.1186/s12917-022-03527-7)
Supplement: Supplementary file 1 — Additional file 1: Table 1. Information on the 14 cats as negative controls in the neutralization test. [file 12917_2022_3527_MOESM1_ESM.docx]

**Supplementary Figure1.**

**Transition of COVID-19 cases from 16 January to 31 August, 2020 reported in the national surveillance in Japan.** The cumulative COVID-19 cases on our sampling completion date (i.e., 31 July, 2020) and those on the sampling completion date of the previous study (i.e., June, 2020) are shown as double arrows at the bottom of the graph.

**

**
