# Supplementary material for: Guest edited collection serological study of SARS-CoV-2 antibodies in japanese cats using protein-A/G-based ELISA
Source: BMC Vet Res. 2022 Dec 21;18:443. doi: 10.1186/s12917-022-03527-7 (PMC9767852; doi:10.1186/s12917-022-03527-7)
Supplement: Supplementary file 2 — Additional file 2: Figure 1.Transition of COVID-19 cases from 16 January to 31 August, 2020 reported in the national surveillance in Japan. [file 12917_2022_3527_MOESM2_ESM.docx]

**Supplementary Figure 3.**

**Correlation of IgG reactivities obtained by protein-A/G-based ELISA with those obtained by anti-feline-IgG-based ELISA.** IgG reactivity to the receptor-binding domain (RBD) protein was measured by protein-A/G-based ELISA. The cutoff was set as the mean value + 3 SDs of negative control samples (dashed line). Samples with a higher value than the cutoff were considered positive. The circles indicate individual tested samples. The star indicates a sample with SARS-CoV-2 neutralizing activity. We examined 34 samples collected, including 9 positive and 25 negative samples in the protein-A/G-based ELISA, detecting the anti-S1-protein IgG antibody, and 43 negative control samples. The data generated by protein-A/G-based ELISA and conventional anti-feline-IgG-based ELISA were analyzed using a Pearson correlation coefficient test. A strong correlation (*r*^2^ = 0.97 and *p* < 0.0001) was detected. The regression line and 95% confidence intervals are indicated by the black line and gray area, respectively.
