# Supplementary material for: Guest edited collection serological study of SARS-CoV-2 antibodies in japanese cats using protein-A/G-based ELISA
Source: BMC Vet Res. 2022 Dec 21;18:443. doi: 10.1186/s12917-022-03527-7 (PMC9767852; doi:10.1186/s12917-022-03527-7)

**Supplementary Figure 2.**

**Binding ability of protein-A/G conjugated with horse radish peroxidase to feline IgG and IgM, and rabbit IgG.** Protein A/G conjugated with horseradish peroxidase was reacted with cat IgG (No. 002-0102, Rockland Immunochemicals, Pottstown, PA, USA), cat IgM (No. 002-0107, Rockland Immunochemicals), rabbit IgG (No. 148-09551, Fujifilm Wako, Tokyo, Japan), and bovine serum albumin (BSA, Nacalai Tesque, Kyoto, Japan). Each immunoglobulin and BSA were repeatedly diluted 10-fold in 0.2mol/L phosphate buffer solution (pH 7.6) from 100 mg/ml to 1 × 10^–9^ mg/ml, and 100 μL of the solution were incubated at 4 °C overnight. The plates were washed once and incubated with protein A/G conjugated with horseradish peroxidase, which was diluted in phosphate buffer saline (PBS) containing 0.05% Tween-20 (PBS-T) (1:25,000; Thermo Fisher Scientific, Waltham, MI, USA) at 25 °C for 1 h. After washing 3 times, the plates were incubated with *o*-phenylenediamine dihydrochloride (Merck, Darmstadt, Germany) at 25 °C for 30 min. The reaction was terminated by adding 4 N H_2_SO_4_. The absorbance at 492 mm (A_492_) was measured using a spectrophotometer (Multiskan JX; Thermo Fisher Scientific). All samples were assayed in parallel on the same plate. All experiments were performed in triplicate. PBS-T was used in all washes. Feline IgG and rabbit IgG could be detected at 1 × 10 ^–6^ mg/mL or higher, but rabbit IgG reacted more strongly with protein-A/G than feline IgG. Feline IgM could be detected at 1 × 10^–2^ mg/ml or higher, and BSA could not be detected up to 100 mg/mL.


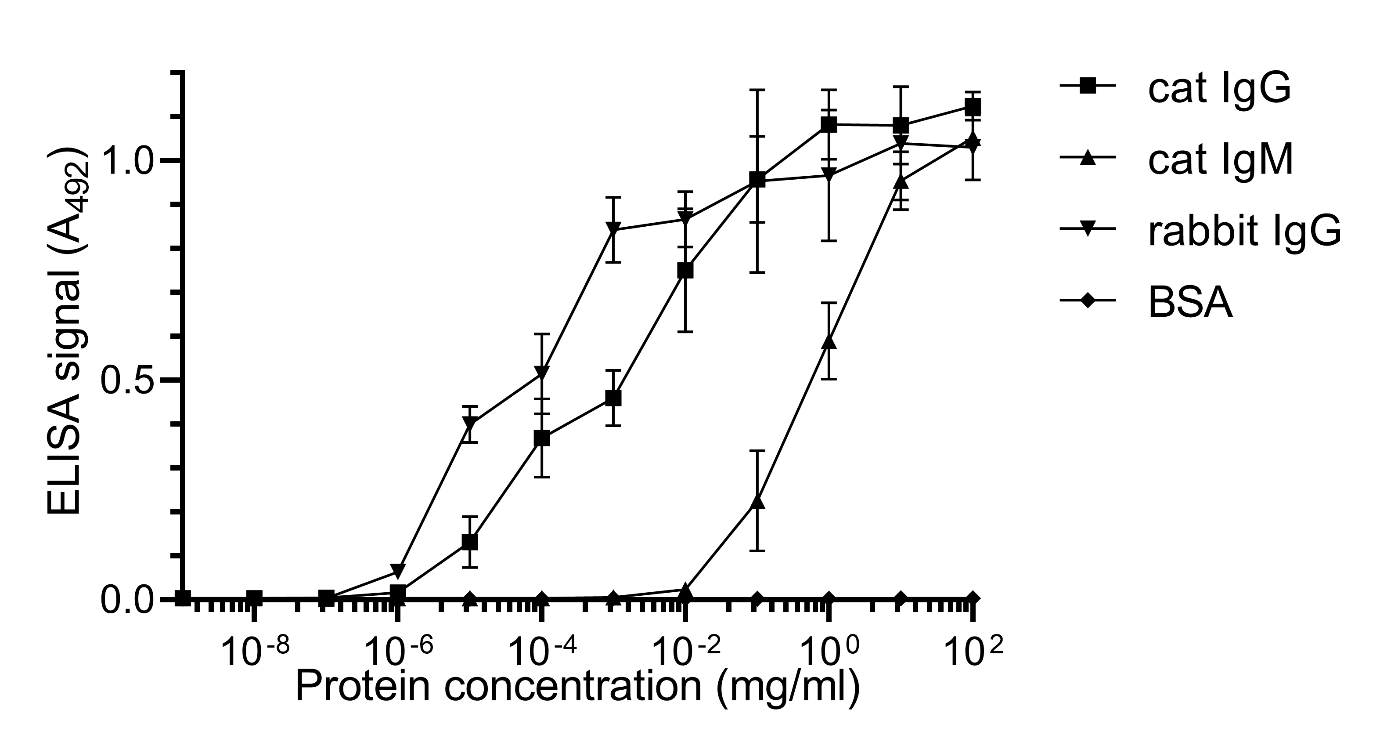

Supplement: Supplementary file 3 — Additional file 3: Figure 2. Binding ability of protein-A/G conjugated with horse radish peroxidase to feline IgG and IgM, and rabbit IgG. [file 12917_2022_3527_MOESM3_ESM.docx]
