# Supplementary material for: Guest edited collection serological study of SARS-CoV-2 antibodies in japanese cats using protein-A/G-based ELISA
Source: BMC Vet Res. 2022 Dec 21;18:443. doi: 10.1186/s12917-022-03527-7 (PMC9767852; doi:10.1186/s12917-022-03527-7)
Supplement: Supplementary file 4 — Additional file 4: Figure 3. Correlation of IgG reactivities obtained by protein-A/G-based ELISA with those obtained by anti-feline-IgG-based ELISA. [file 12917_2022_3527_MOESM4_ESM.docx]

**Supplementary information**

**Supplemental Table and Figures**

Supplementary Table 1. **Information of the 14 cats as negative controls in the neutralization test**

Supplementary Figure 1; **The transition of COVID-19 cases, from 16 January to 31 August, 2020, reported in the national surveillance in Japan**

Supplementary Figure 2. **Binding ability of protein-A/G conjugated with horseradish peroxidase to feline IgG and IgM, and rabbit IgG**

Supplementary Figure 3. **Correlation of IgG reactivities obtained by protein-A/G-based ELISA with those obtained by anti-feline-IgG-based ELISA**
